# Systemic effects induced by intralesional injection of ω-conotoxin MVIIC after spinal cord injury in rats

**DOI:** 10.1186/1678-9199-20-15

**Published:** 2014-04-16

**Authors:** Karen M Oliveira, Carla Maria O Silva, Mário Sérgio L Lavor, Isabel R Rosado, Fabíola B Fukushima, Anna Luiza FV Assumpção, Saira MN Neves, Guilherme R Motta, Fernanda F Garcia, Marcus Vinícius Gomez, Marília M Melo, Eliane G Melo

**Affiliations:** 1 Departamento de Clínica e Cirurgia Veterinária, Escola de Veterinária, Universidade Federal de Minas Gerais, Avenida Antônio Carlos, 6627, Pampulha, Belo Horizonte, MG CEP 30123-970, Brasil; 2 Departament of Agrarian and Environmental Sciences, State University of Santa Cruz, Ilhéus, Bahia State, Brazil; 3 National Institute of Sciences and Technology on Molecular Medicine, School of Medicine, Federal University of Minas Gerais, Belo Horizonte, Minas Gerais State, Brazil

**Keywords:** *Conus magus*, Cone snail, Histopathology, Hematology, Biochemistry

## Abstract

**Background:**

Calcium channel blockers such as conotoxins have shown a great potential to reduce brain and spinal cord injury. MVIIC neuroprotective effects analyzed in *in vitro* models of brain and spinal cord ischemia suggest a potential role of this toxin in preventing injury after spinal cord trauma. However, previous clinical studies with MVIIC demonstrated that clinical side effects might limit the usefulness of this drug and there is no research on its systemic effects. Therefore, the present study aimed to investigate the potential toxic effects of MVIIC on organs and to evaluate clinical and blood profiles of rats submitted to spinal cord injury and treated with this marine toxin. Rats were treated with placebo or MVIIC (at doses of 15, 30, 60 or 120 pmol) intralesionally following spinal cord injury. Seven days after the toxin administration, kidney, brain, lung, heart, liver, adrenal, muscles, pancreas, spleen, stomach, and intestine were histopathologically investigated. In addition, blood samples collected from the rats were tested for any hematologic or biochemical changes.

**Results:**

The clinical, hematologic and biochemical evaluation revealed no significant abnormalities in all groups, even in high doses. There was no significant alteration in organs, except for degenerative changes in kidneys at a dose of 120 pmol.

**Conclusions:**

These findings suggest that MVIIC at 15, 30 and 60 pmol are safe for intralesional administration after spinal cord injury and could be further investigated in relation to its neuroprotective effects. However, 120 pmol doses of MVIIC may provoke adverse effects on kidney tissue.

## Background

Spinal cord injury (SCI) is a leading cause of permanent disability in young adults [1-3]. At the time of trauma, the primary lesion usually leads to the disruption of axons, neurons and neuroglia cell bodies, resulting in nerve impulses interruption. Afterwards, the secondary neurodegenerative events start and worsen the initial injury. Excessive accumulation of intracellular calcium is a common phenomenon after SCI and it is the most critical step in ionic dysregulation that generates axonal injury and eventual apoptosis or necrosis via an increase in cellular enzymes activation, mitochondrial damage, acidosis, and free radicals production [4-9].

Calcium channel blockers (CCB) have shown great potential in reducing brain and spinal cord injury, by preventing the intense ion influx and, consequently, the secondary injury progression [10,11]. A wide variety of natural CCB were identified in animal venoms containing neuroactive or neuroprotective peptides, including the conotoxins from *Conus* snails [12]. Omega-conotoxin MVIIC (MVIIC) is a member of the CCB toxin family constituted by 26 aminoacids [13]. It selectively inhibits the types N (Ca_v_2.1), P, and Q (Ca_v_2.2) voltage-dependent calcium channels (VDCC) that are essential in the release of neurotransmitters [14-16]. In recent years, studies on MVIIC effects have shown that it significantly reduces calcium influx through VDCC in several *in vitro* models of ischemic brain and spinal injuries [15,17-21]. Thus, these findings suggest a potential role of MVIIC in preventing secondary injuries after spinal trauma.

However, early clinical experience with MVIIC demonstrates that side effects may limit the usefulness of this class of drugs [22,23]. Envenomation by *Conus* toxins is characterized by various symptoms such as intense pain followed by shaking, generalized paresthesia, neuromuscular paralysis and death caused by respiratory failure [23,24]. No information about the pharmacokinetics and pharmacodynamic characteristics of MVIIC is available. To be possible to evaluate the effects of toxin on SCI, safety should be the overriding principle in the selection for the best dose. The present study was designed to determine, for the first time, the *in vivo* effects of MVIIC on blood profile and histopathological changes that it may provoke. In this experiment, MVIIC was intralesionally injected due to its peptide nature, so the toxin was directly administered in the central nervous system (CNS), and additionally this route enables local treatment, requiring a smaller amount of toxin with fewer side effects [25,26].

## Methods

### Experimental design

Thirty adult male Wistar rats weighting 400 to 450 g were randomly distributed into five groups. Rats were housed in a controlled environment and provided with commercial rodent food and water *ad libitum*. The Ethics Committee on Animal Experimentation of the Federal University of Minas Gerais (CETEA/UFMG) approved the present study under protocol n. 075/10. All animals experiments followed the recommendations of Guide for the Care and Use of Laboratory Animals of the US National Institute of Health.

Animals were premedicated with tramadol chloride (4 mg/kg, subcutaneously), and anesthesia was induced and maintained with isoflurane in a non-rebreathing circuit, through a facemask. The animals were positioned in a stereotaxic apparatus, received prophylactic antibiotic with cephalotin (60 mg/kg, subcutaneously) and then, prepared for asseptic surgery. An incision was made in the dorsal midline skin and subcutaneous tissues from T8 to L1, and the muscle and tissue overlying the spinal column were blunt-dissected away revealing the laminae. Using the spiny process of T13 as a landmark, laminectomy of T12 was performed with a pneumatic drill and the lamina was carefully removed to expose the spinal cord. Extradural compression of the spinal cord at the vertebral level of T12 was achieved as previously described [27-30] using a weight of 70 g/cm^2^. Five minutes later, an intralesional injection was performed according to the experimental protocol. The incision was closed in two layers and the animals were allowed to recover from anesthesia in a warmed (37°C) box.

Post-operative care procedures involved manual expression of the bladder, three times a day, tramadol chloride (2 mg/kg, orally, every eight hours) for three days, and cephalexin (30 mg/kg, orally, twice a day) for five days.

### Pharmacological treatment

Five minutes after the incision, 2 μL of treatment was administered into the injury center using a Hamilton microsyringe, as previously described [31]. The animals were distributed into five groups, with six rats each, according to the treatment protocol: placebo treatment with sterile water (PLA), 15 pmol of MVIIC (G15), 30 pmol of MVIIC (G30), 60 pmol of MVIIC (G60), and 120 pmol of MVIIC (G120). Doses were based on studies describing that MVIIC doses of 3 pmol have analgesic effects by blocking the VDCC type P/Q, and those of 100 pmol had side effects [23,32].

For eight days, experiments focused on clinical observation. All animals were euthanized at Day 8 following SCI. Clinical and histopathological evaluations were carried out by investigators who did not take part into the research.

### Clinical evaluation

For three days before SCI, animals were allowed to adapt to the open field arena – that had 90 cm in diameter and three inches high – for 15 minutes to clinical evaluation. After trauma, they were monitored for the presence of widespread tremor, walking in circle or muscle weakness, during the first five hours after the toxin administration and daily until euthanasia [33].

### Blood collection

Blood samples were collected prior to experiment and eight days after treatment by caudal vein puncture in two types of tubes, with anticoagulant sodium fluoride in order to access hematologic profile, and without anticoagulant to collect serum and evaluate biochemical profiles, both analyzed immediately.

### Hematological parameters

Hematological parameters including red blood cells (RBC), white blood cells (WBC) and hemoglobin concentration (Hb) were determined using Abacus Junior Vet electronic counter (Diatron Messtechnik GmbH, Austria). The RBC indices – namely mean corpuscular volume (MCV), mean corpuscular hemoglobin (MCH) and mean corpuscular hemoglobin concentration (MCHC) – were calculated using the RBC count, Hb and hematocrit (Ht) values. Blood smears were prepared on glass slides (26 × 79 mm), fixed with May-Grunwald solution and stained with Giemsa in order to carry out differential counting of leukocytes and total number of platelets [34]. Volume of packed RBC or Ht was determined using a microhematocrit centrifuge (Model Spin 1000®, Micro Spin, USA). The blood was centrifuged to obtain plasma and to determinate total protein by refractometry.

### Biochemical parameters

Urea, creatinine, alanine aminotransferase (ALT) and aspartate aminotransferase (AST) were determined with the aid of commercial kits from Synermed® (Westfield, USA) and Cobas Mira Classic® chemical analyzer (Global Medical Instrumentations, USA).

### Histological analysis of tissue injury

On Day 8 after surgery, the rats were deeply anesthetized with an overdose of sodium thiopental (100 mg/kg), intraperitoneally. The animals were perfused with 300 mL of 0.9% sodium chloride saline followed by 300 mL of 10% phosphate-buffered formalin (pH 7.4). Following perfusion, the brain, heart, liver, kidney, lung, spleen, stomach, lumbar muscle, intestine, pancreas and adrenal were removed and placed overnight in 10% phosphate-buffered formalin. Twenty-four hours later, organ samples were dehydrated in a series of alcohol grades and embedded in paraffin wax. Briefly, 4-μm thick longitudinal sections were stained with routine hematoxylin-eosin (HE) for pathological studies.

Lesion areas were classified in nine grades, according to the histological pattern of intensity (mild, moderate, and severe) and extension (focal, multifocal, and diffuse) of the lesion (Table 1).

**Table 1:** Bladder scores according to the lesion histological pattern

**Scores**	**Histological pattern**	
	**Intensity**	**Extension**
**1**	Mild	Focal
**2**	Mild	Multifocal
**3**	Mild	Diffuse
**4**	Moderate	Focal
**5**	Moderate	Multifocal
**6**	Moderate	Diffuse
**7**	Severe	Focal
**8**	Severe	Multifocal
**9**	Severe	Diffuse

### Statistical analysis

All collected data were analyzed using Prism 5® for Windows (GraphPad Software, La Jolla, USA) and were expressed as mean ± standard deviation (SD). Normal distributed data were subjected to analysis of variance (ANOVA), followed by *t*-paired test between times and Student-Newman-Keuls test between groups. Non-parametric parameters were subjected to Kruskall-Wallis test and Dunn’s post hoc test (*p* < 0.05).

## Results and discussion

In the current experiment, we studied the clinical, hematological, biochemical and tissue histopathological changes to investigate any toxic effects caused by MVIIC. These parameters help to identify possible changes caused by such toxins, the severity of the alterations and which doses can be clinically used in SCI.

### Clinical evaluation

Although frequently reported, in the present study no dose provoked muscle weakness or paralysis as seen by Dalmolin *et al*. [23] at 100 or 300 pmol by intrathecal (IT) route or even shaking at 10 pmol by intracerebroventricular (IC) route. These differences may be attributable to the fact that it was impossible to evaluate variables such as hindlimb locomotor deficit, flaccid paralysis or decreased tail withdrawal response, describe by these authors, because they are similar to clinical signs of SCI. Besides, Malmberg and Yaksh [33] noticed that body shaking occurred 30 minutes after the injection and, at this time, the animal was recovering from anesthesia. Moreover, Dalmolin *et al*. [23] observed that IC route had more side effects than IT. This fact could be associated to the high density of P/Q-type expressed in nociceptive pathways at the supraspinal site, which emphasizes the powerful effect of MVIIC injected by IC route [35]. It also could be inferred that due to the IL injection into the spinal cord, we did not notice even body shaking as seen at 10 pmol by IC route.

### Hematological parameters

**Figure 1:**
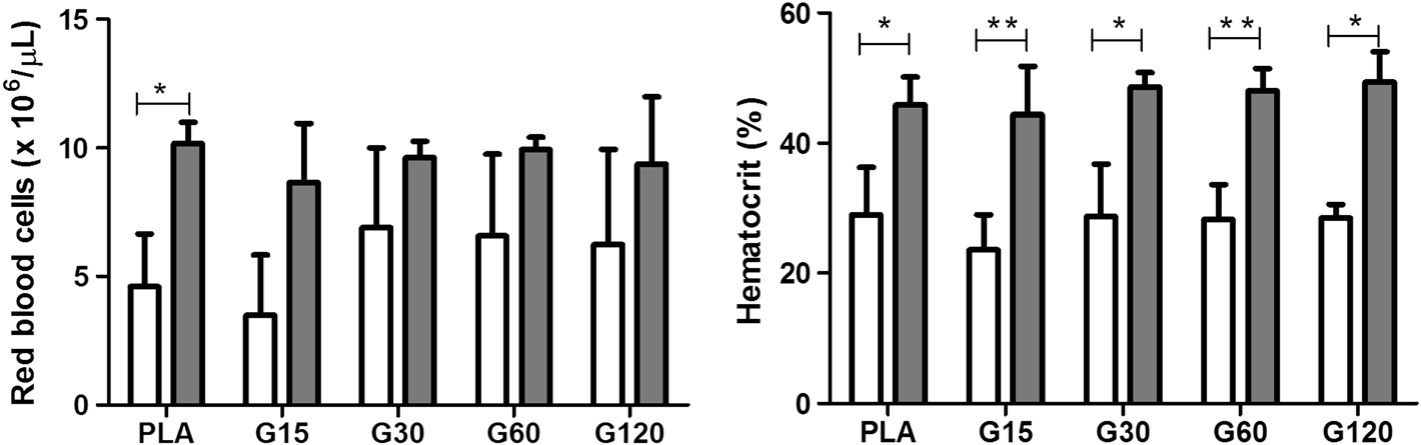
**Effects of different doses of MVIIC on red blood cells (× 10**^**6**^**/μL) and hematocrit.** Mean number ± SD of red blood cells and hematocrit of rats submitted to compressive spinal cord injury and treated with placebo (PLA, positive control) or ω-conotoxin MVIIC (G15, 15 pmol MVIIC; G30, 30 pmol MVIIC; G60, 60 pmol MVIIC), preoperatively (white column) and eight days after treatment (gray column) (*p < 0.05; **p < 0.01). Red blood cells and hematocrit normal values are, respectively, 7.62 - 9.99 × 10^6^/μL and 38.5-52%, as described by Giknis and Clifford [39].

**Figure 2:**
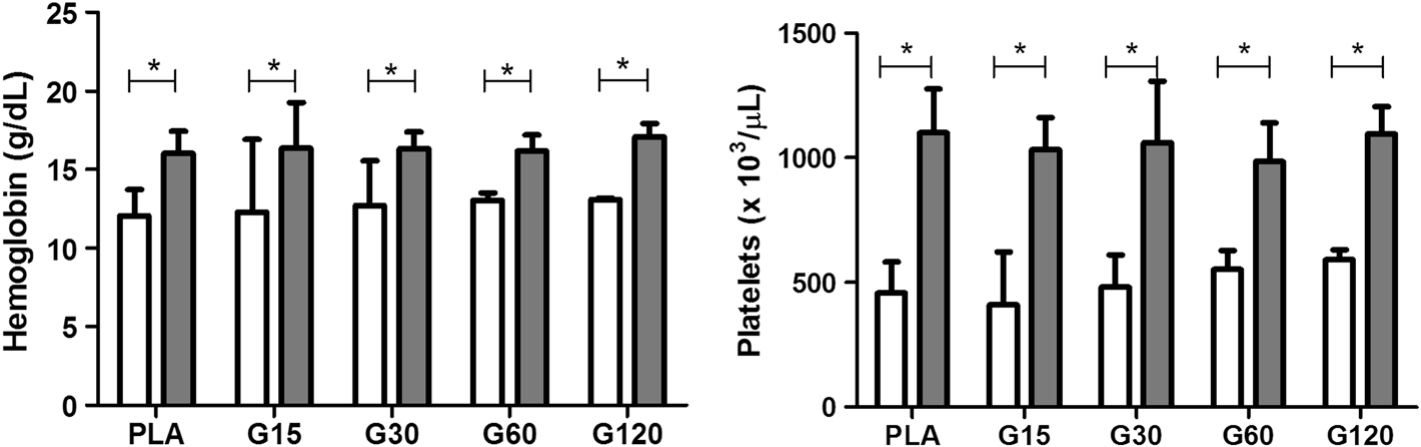
**Effects of different doses of MVIIC on hemoglobin (g/dL) and platelets (× 10**^**3**^**/μL).** Mean number ± SD of hemoglobin and platelets of rats submitted to compressive spinal cord injury and treated with placebo (PLA, positive control) or ω-conotoxin MVIIC (G15, 15 pmol MVIIC; G30, 30 pmol MVIIC; G60, 60 pmol MVIIC), preoperatively (white column) and eight days after treatment (gray column) (*p < 0.05). Hemoglobin and platelets concentration normal values are, respectively, 13.5-17.4 g/dL and 574–1253 × 10^3^/μL, as described by Giknis and Clifford [39].

In this experiment, an increase in RBC, Ht (Figure 1) and Hb (Figure 2) concentration in all groups may be due to the postoperative stress, restraint and anesthesia prior to euthanasia, leading to a splenic contraction and consequent erythrocyte release [36]. Furthermore, hemoconcentration is most likely a result of the liquid loss due to the weakened condition of rats in the postoperative period and reduced water consumption. Dehydration was not clinically observed, but water intake was reduced as suggested the levels of bottles. Moreover, in the dehydration process, total protein concentration is augmented [37]. Proteins have many functions in the organism and their levels helps in the diagnosis and prognosis of hydration status, inflammation, infection, nutritional status and changes in protein synthesis [38]. In this study, the absolute values of total protein showed no differences among times and groups (Additional file 1), suggesting that hematological changes are likely related to postoperative stress and euthanasia. Nevertheless, these parameters remained close to the reference values for the species [39].

The values of MCV (Additional file 2), MCH (Additional file 3) and MCHC (Additional file 4) did not differ among times or groups, remaining within the physiologic patterns of species.

The increase of platelets (Figure 2) is a common finding in patients who underwent surgical intervention probably by tissue damage and inflammation. The relevance of this event is not clear yet [40-42]. Despite this fact, the parameters were within physiological standards for these animals (574–1253 × 10^3^ cells/μL) [39].

There are no reports of hematologic evaluation in animals that receive MVIIC, and this study shows that it does not cause anemia, hemolysis, vascular changes or interference in the hematologic response when applied by intralesional route.

**Figure 3:**
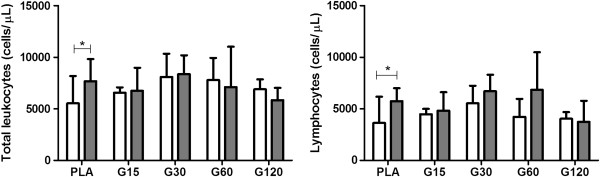
**Effects of different doses of MVIIC on total leukocytes (cells/μL) and lymphocytes (cells/μL).** Mean number ± SD of total leukocytes and lymphocytes of rats submitted to compressive spinal cord injury and treated with placebo (PLA, positive control) or ω-conotoxin MVIIC (G15, 15 pmol MVIIC; G30, 30 pmol MVIIC; G60, 60 pmol MVIIC), preoperatively (white column) and eight days after treatment (gray column) (*p < 0.05). Total leukocyte and lymphocyte normal values are, respectively, 1980–11060 cells/μL and 1190–9450 cells/μL, as described by Giknis and Clifford [39].

There was a significant increase of the total leukocyte count in the PLA (7.680 ± 2.140 cells/μL) among the times of collection, not exceeding the maximum limits for the species (Figure 3) (*p* < 0.05). The leukocytosis can be attributed to stress induced by physical restraint at the time of euthanasia. In acute stress conditions, the release of endogenous glucocorticoids promotes increased blood and lymph circulation so leukocytes pass into the peripheral blood causing physiological leukocytosis [43]. However, only the PLA group showed this significant leukocyte increase. Another possible explanation is that in animals with spinal cord trauma, there is great local inflammatory reaction in PLA group. In contrast, MVIIC groups did not show significant leukocyte augmentation and we can infer a possible anti-inflammatory effect, requiring further investigation.

There was no change in the absolute number of monocytes (Additional file 5), neutrophils (Additional file 6), and basophils (Additional file 7) among groups or times. Regarding the number of lymphocytes, it can be observed a significant increase (*p* < 0.05) in animals of PLA group (5.750 ± 1.250) eight days after the injury (Figure 3). Lymphocytes are the major circulating cells of rats, so the leucocytosis is probably due to lymphocytosis. Moreover, there was no difference among times and groups related to the absolute number of eosinophils, showing that MVIIC did not cause sensitization processes indicated by eosinophilia [44].

As seen before, there was no toxic effect of treatment and, therefore, the toxin at the tested doses did not cause any hematological change. These results are unprecedented in the literature.

### Biochemical parameters

**Figure 4:**
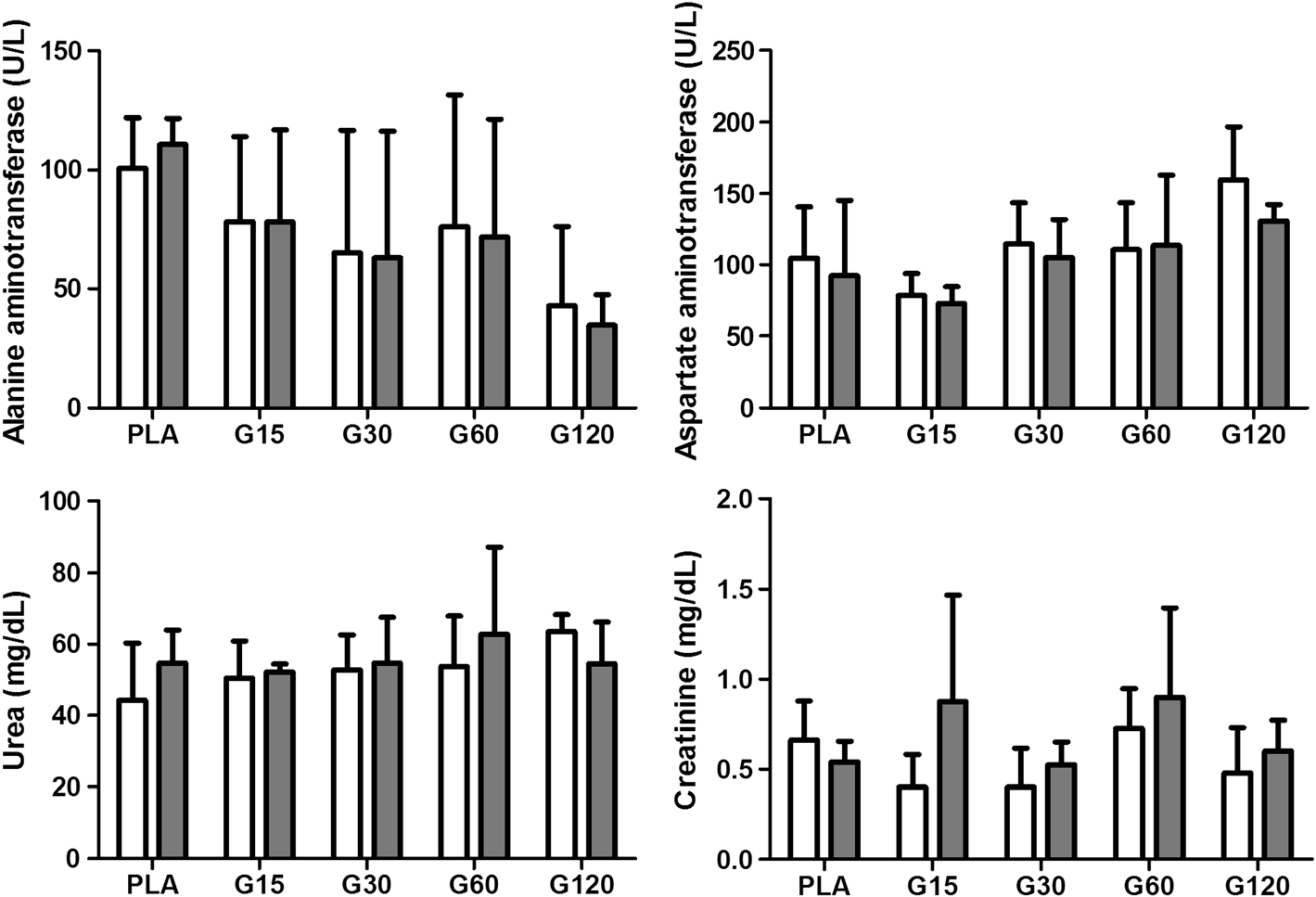
**Effects of different doses of MVIIC on biochemical parameters.** Mean number ± SD of alanine aminotransferase (ALT) (U/L), aspartate aminotransferase (AST) (U/L), urea (mg/dL) and creatinine (mg/dL) in different times (preoperatively and eight days after injection) (*p <* 0.05). Controls were injected with sterile water (PLA) and other groups received different doses of MVIIC (15, 30, 60 and 120 pmol). ALT, AST, urea and creatinine normal values are, respectively, 19–48 U/L, 63–175 U/L, 10.7-20 mg/dL and 0.3-0.5 mg/dL, as described by Giknis and Clifford [39].

Biochemical analysis is an important variable for evaluation in studies on toxins which reflects hepatic and renal function. Some enzymes such as AST and ALT are used to evaluate liver function, a key organ for drug metabolism. They reveal abnormalities, and deleterious effects of toxins may increase their levels, especially ALT, which is more specific for liver changes in rats [45]. In our findings, there was no difference in the values of AST and ALT among time points (Figure 4), so there was no toxic effect on liver tissue, which was confirmed by liver histopathology. In addition to reflecting liver abnormalities, AST is used to assess myocardial infarction. Thus, according to our results, it can be stated that there was no significant cardiac muscle damage confirmed by heart histopathology.

Furthermore, renal excretion is a major route for drug elimination and their commitment may be evidenced by increases in serum creatinine and urea, indicators of glomerular filtration. Creatinine is considered a more reliable indicator that may be affected by the influence of extrarenal factors, and it is the final product of energy used by muscle tissue [46]. Its concentration in blood depends on muscle injury, physical efforts and also meat intake in the case of carnivorous. It is filtered by the renal glomeruli, so renal glomerular lesions are verified by increased creatinine and urea [46,47]. These values did not differ among groups and times (Figure 4). However, high levels of urea and creatinine in comparison with the physiological data set by Giknis and Clifford [39] were noticed, both preoperatively and on the eighth day after trauma. Therefore, different standard physiological data from the literature were observed.

### Histopathological evaluation

Histopathological studies revealed no significant abnormalities in tissue of brain, lung, heart, liver, adrenal, muscle, pancreas, spleen, and stomach in all groups.

**Figure 5:**
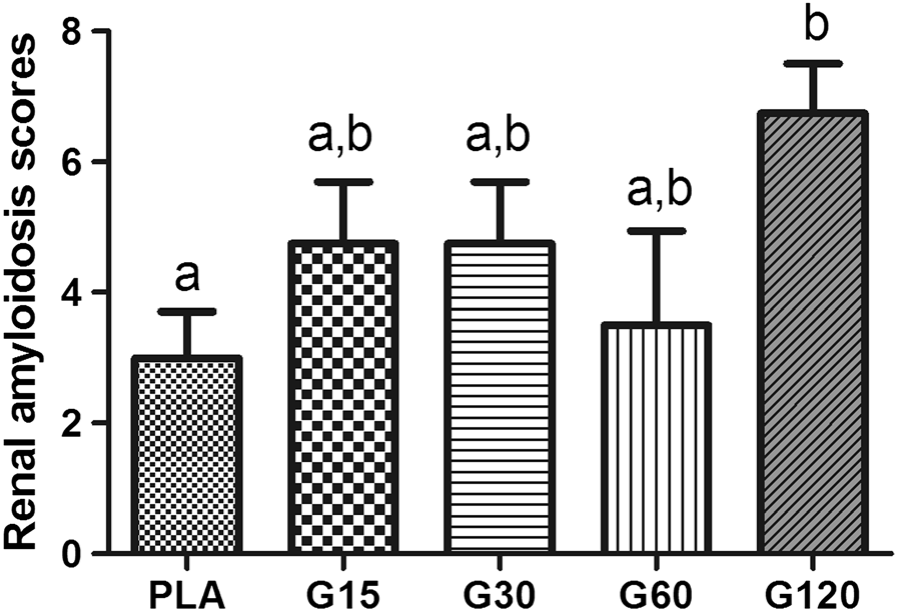
**Median scores for renal amyloidosis in rats submitted to spinal cord injury.** Controls were injected with sterile water (PLA) and other groups received different doses of MVIIC (15, 30, 60 and 120 pmol). Lowercase letters express statistically differences among groups, after eight days of spinal cord injury (Kruskal-Wallis test and Dunn’s post hoc test; *p* < 0.05).

**Figure 6:**
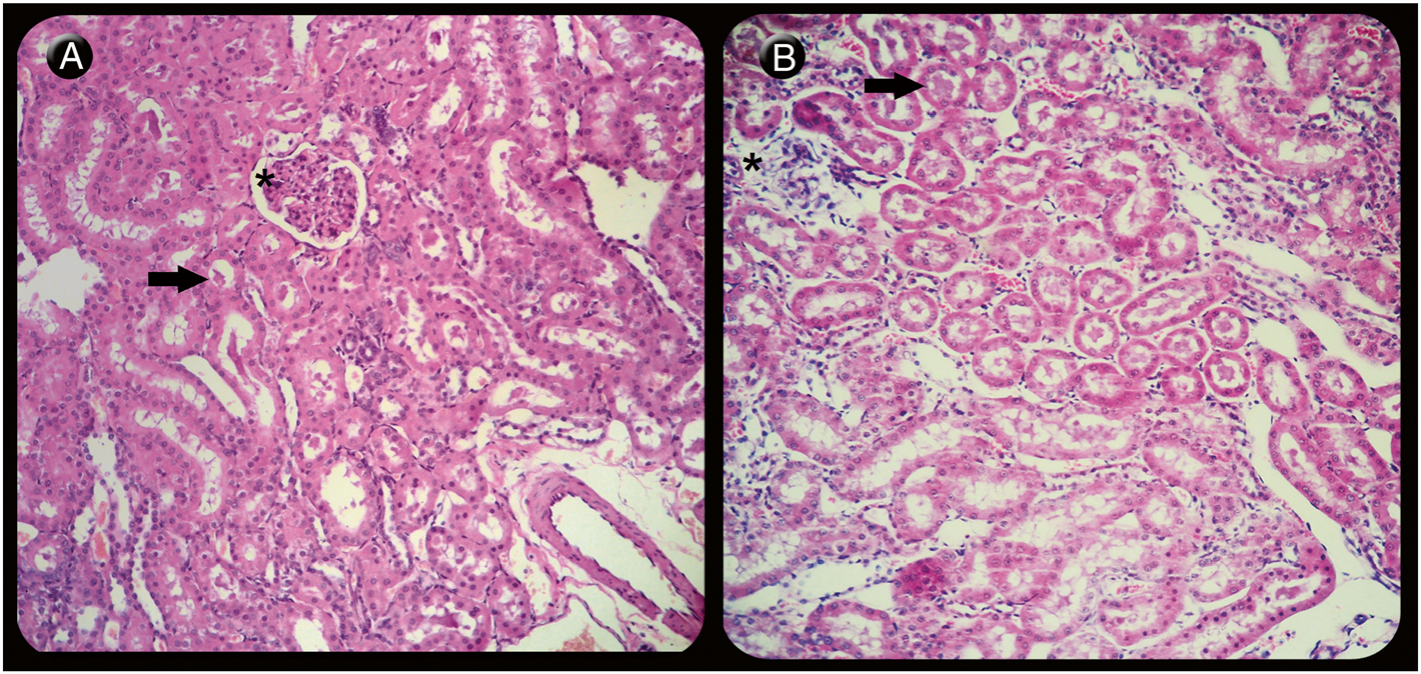
**Light microscopy of renal longitudinal sections of rats stained with hematoxylin-eosin.(A)** Kidney showing mild degenerative changes in tubules (arrow) and glomeruli (asterisk) in the control group (PLA) (210×) when compared to **(B)** the group that received 120 pmol of MVIIC (G120), revealing severe degeneration in the Bowman’s space, glomerular (asterisk) and tubular epithelial cells with deposition of eosinophilic material inside renal tubules (arrow) (207×).

In our study kidney tissues showed degenerative changes, especially when treated with 120-pmol doses of MVIIC, including degeneration in the Bowman’s space, glomerular and tubular epithelial cells with deposition of eosinophilic material (amyloid) inside renal tubules, atrophy and glomerular sclerosis [48]. The 120-pmol dose significantly differed from others groups (*p* < 0.05) (Figures 5 and 6). This type of injury may lead to renal failure and have been observed in animals after spinal trauma following spider or scorpion envenomation [49-51]. It seems that in addition to amyloidosis caused by SCI, the 120-pmol dose intensified the kidney injury, even without increased levels of urea and creatinine, since it occurs when over 75% of renal function is lost [47]. Although there is no record of systemic effects of MVIIC, the highest dose increased renal changes, possibly by a direct glomerulopathy to renal tubule, or indirect, inflammatory response, as seen with others drugs [52].

## Conclusion

Our results suggest that MVIIC at 15, 30 and 60 pmol are safe to be used via intralesional route after spinal cord injury. The 120-pmol dose of MVIIC was detrimental to renal tissue, but it was not enough to change the renal function. More studies are necessary to investigate other routes of MVIIC administration and its possible side effects.

### Ethics committee approval

All procedures of the current research were performed in accordance with ethical principles of animal experimentation adopted by the Ethics Committee on Animal Experimentation of the Federal University of Minas Gerais (CETEA/UFMG), which approved the present study under protocol n. 075/10.

## Abbreviations

SCI: Spinal cord injury; MVIIC: ω-conotoxin MVIIC; CCB: Calcium channel blockers; VDCC: Voltage-dependent calcium channels; PLA: Placebo; G15: 15 pmol of MVIIC; G30: 30 pmol of MVIIC; G60: 60 pmol of MVIIC; G120: 120 pmol of MVIIC; RBC: Red blood cells; WBC: White blood cells; Hb: Hemoglobin; MCV: Mean corpuscular volume; MCH: Mean corpuscular hemoglobin; MCHC: Mean corpuscular hemoglobin concentration; HT: Hematocrit; ALT: Alanine aminotransferase; AST: Aspartate aminotransferase; HE: Hematoxylin-eosin; SD: Standard deviation.

## Competing interests

The authors declare that there are no competing interests.

## Authors’ contributions

KMO was the main responsible, participated in all stages of the experiment that was part of her master's project. CMOS contributed to the all surgeries and care for animals. MSLL participated in the study design, contributed to the surgeries and helped to draft the manuscript. IRR contributed to some surgeries and care of animals. FBF participated in the study design, contributed to the surgeries and helped to draft the manuscript. ALVFA took care of the animals, assisting with medications, bladder massage and clinical evaluation. SMNN assisted with the microscopic evaluation of tissue. GRM took care of the animals, assisting with medications, bladder massage and clinical evaluation. FFG helped with hematological examinations. MVG participated in the study design and coordination of the project. MMM participated in the study design and helped to draft the manuscript. EGM and MVG participated in the study design, coordination and helped to draft the manuscript. All authors read and approved the final manuscript.

## Supplementary Material

Additional file 1 **Effects of different doses of MVIIC on total protein levels.** After spinal cord injury, controls were injected with sterile water (PLA) and other groups received different doses of MVIIC (15, 30, 60 and 120 pmol). Values represent the means ± SD of six animals at each time. Click here for file

Additional file 2 **Effects of different doses of MVIIC on median corpuscular volume.** After spinal cord injury, controls were injected with sterile water (PLA) and other groups received different doses of MVIIC (15, 30, 60 and 120 pmol). Values represent the means ± SD of six animals at each time. Click here for file

Additional file 3 **Effects of different doses of MVIIC on median corpuscular hemoglobin.** After spinal cord injury, controls were injected with sterile water (PLA) and other groups received different doses of MVIIC (15, 30, 60 and 120 pmol). Values represent the means ± SD of six animals at each time. Click here for file

Additional file 4 **Effects of different doses of MVIIC on mean corpuscular hemoglobin concentration.** After spinal cord injury, controls were injected with sterile water (PLA) and other groups received different doses of MVIIC (15, 30, 60 and 120 pmol). Values represent the means ± SD of six animals at each time. Click here for file

Additional file 5 **Effects of different doses of MVIIC on monocyte levels.** After spinal cord injury, controls were injected with sterile water (PLA) and other groups received different doses of MVIIC (15, 30, 60 and 120 pmol). Values represent the means ± SD of six animals at each time. Click here for file

Additional file 6 **Effects of different doses of MVIIC on neutrophil levels.** After spinal cord injury, controls were injected with sterile water (PLA) and other groups received different doses of MVIIC (15, 30, 60 and 120 pmol). Values represent the means ± SD of six animals at each time. Click here for file

Additional file 7 **Effects of different doses of MVIIC on basophil levels.** After spinal cord injury, controls were injected with sterile water (PLA) and other groups received different doses of MVIIC (15, 30, 60 and 120 pmol). Values represent the means ± SD of six animals at each time. Click here for file
