# Supplementary figures and images for: Systemic effects induced by intralesional injection of ω-conotoxin MVIIC after spinal cord injury in rats
Source: J Venom Anim Toxins Incl Trop Dis. 2014 Apr 16;20:15. doi: 10.1186/1678-9199-20-15 (PMC4021631; doi:10.1186/1678-9199-20-15)

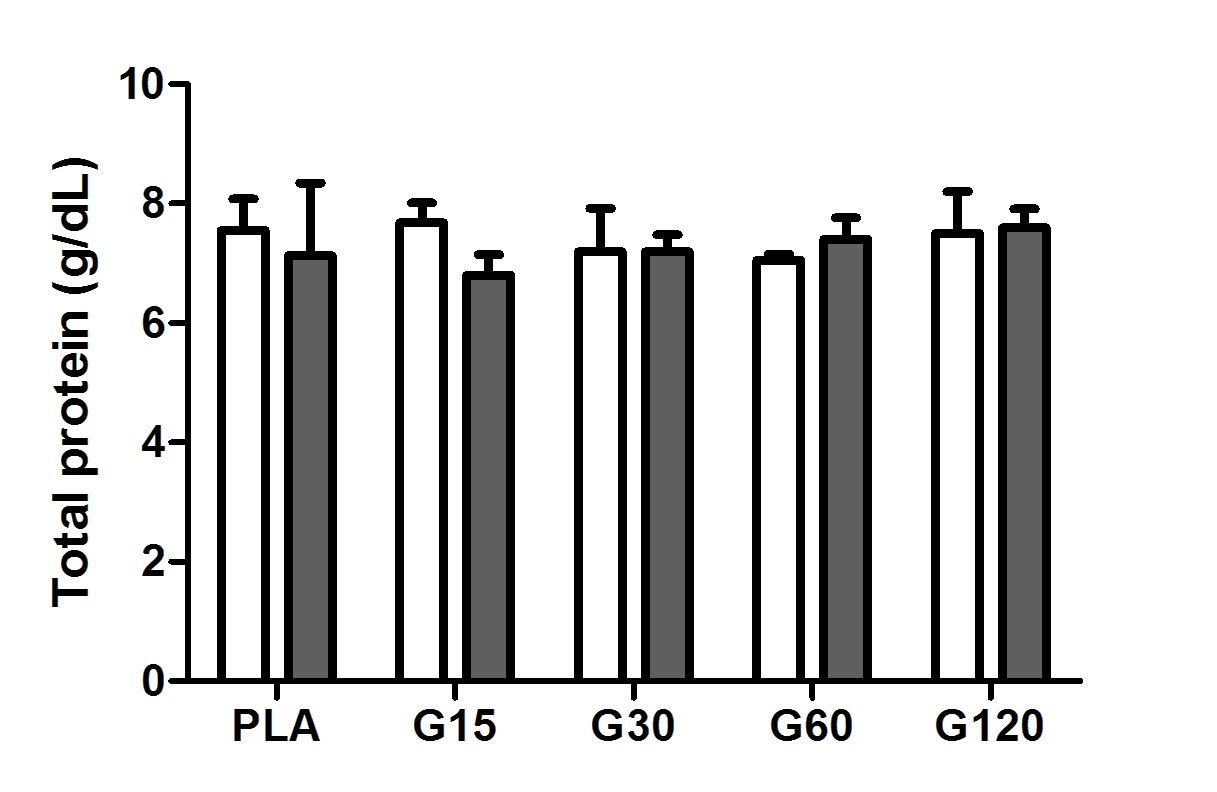

Supplement: Additional file 1 — Effects of different doses of MVIIC on total protein levels. After spinal cord injury, controls were injected with sterile water (PLA) and other groups received different doses of MVIIC (15, 30, 60 and 120 pmol). Values represent the means ± SD of six animals at each time. [file 1678-9199-20-15-S1.tiff]

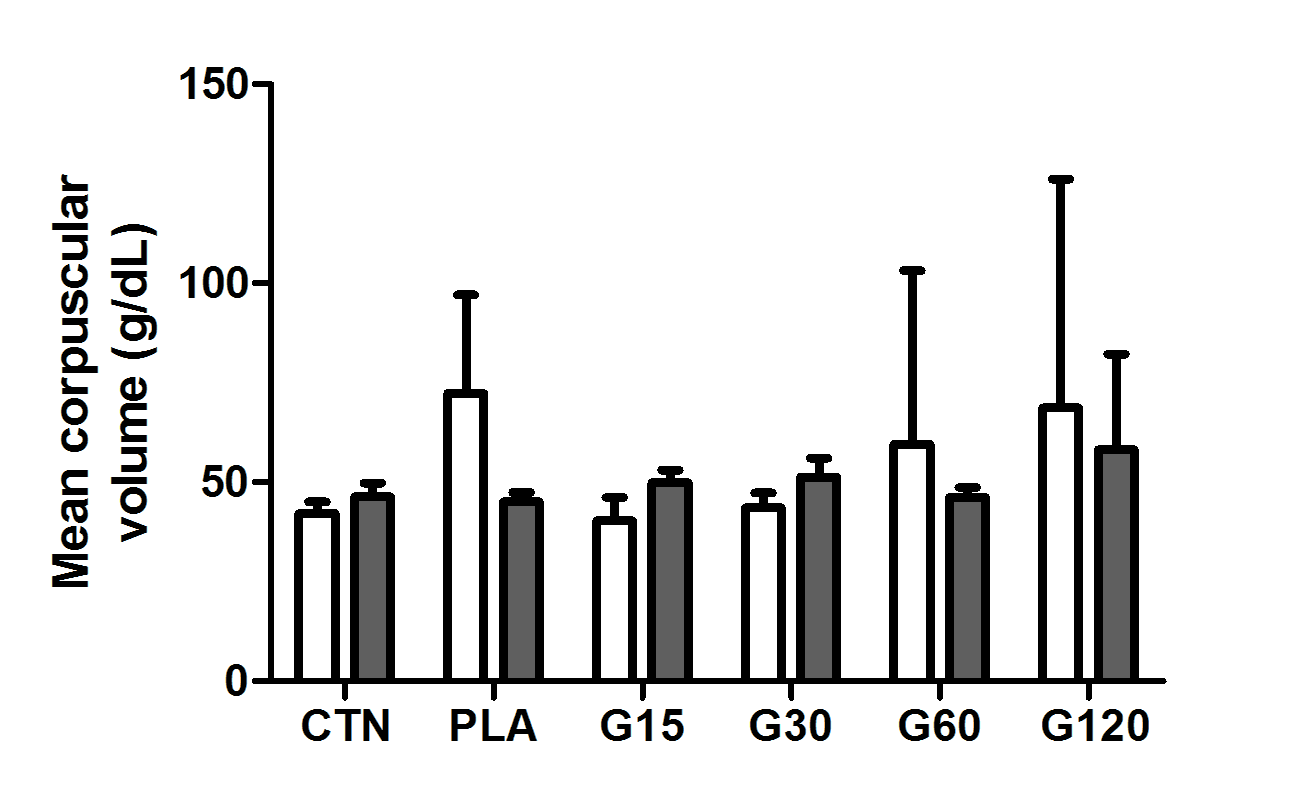

Supplement: Additional file 2 — Effects of different doses of MVIIC on median corpuscular volume. After spinal cord injury, controls were injected with sterile water (PLA) and other groups received different doses of MVIIC (15, 30, 60 and 120 pmol). Values represent the means ± SD of six animals at each time. [file 1678-9199-20-15-S2.tiff]

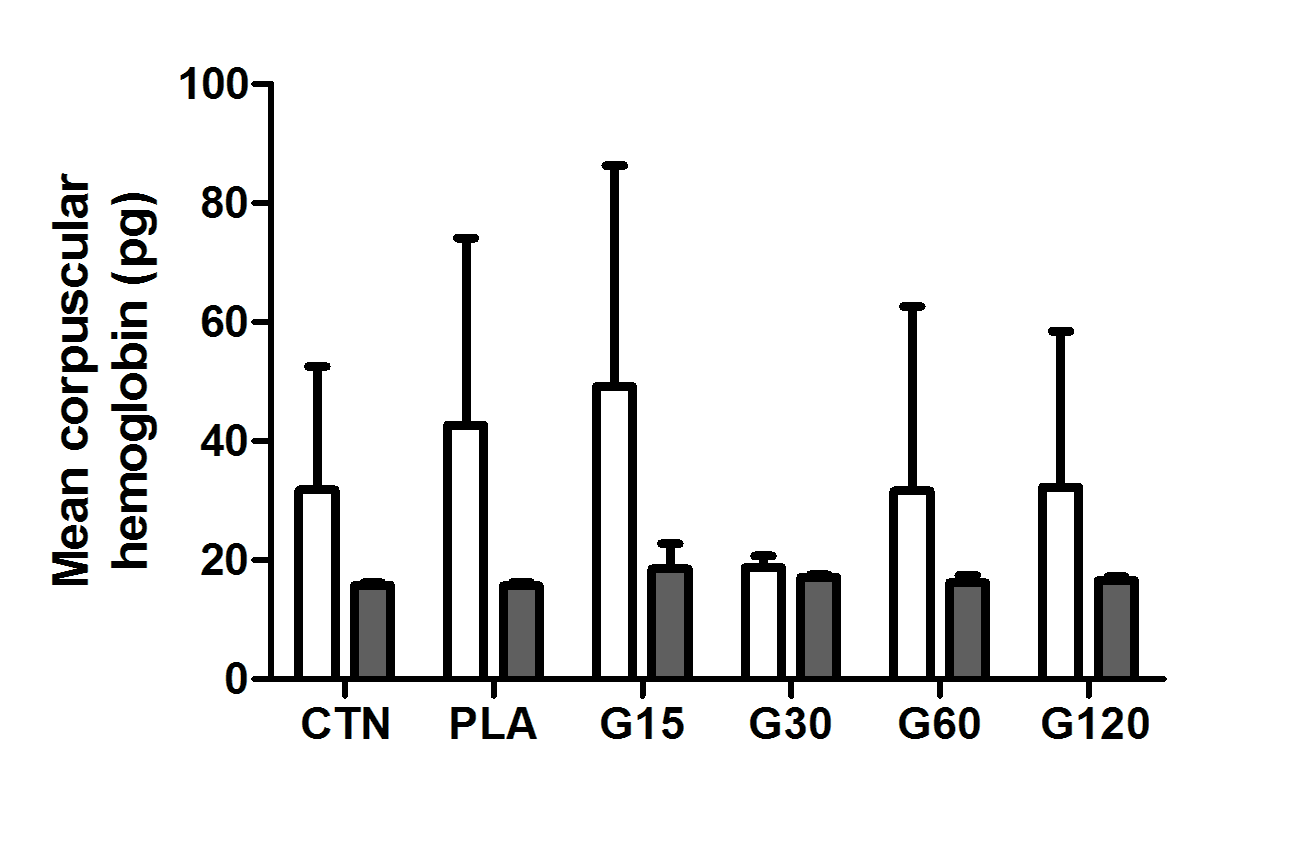

Supplement: Additional file 3 — Effects of different doses of MVIIC on median corpuscular hemoglobin. After spinal cord injury, controls were injected with sterile water (PLA) and other groups received different doses of MVIIC (15, 30, 60 and 120 pmol). Values represent the means ± SD of six animals at each time. [file 1678-9199-20-15-S3.tiff]

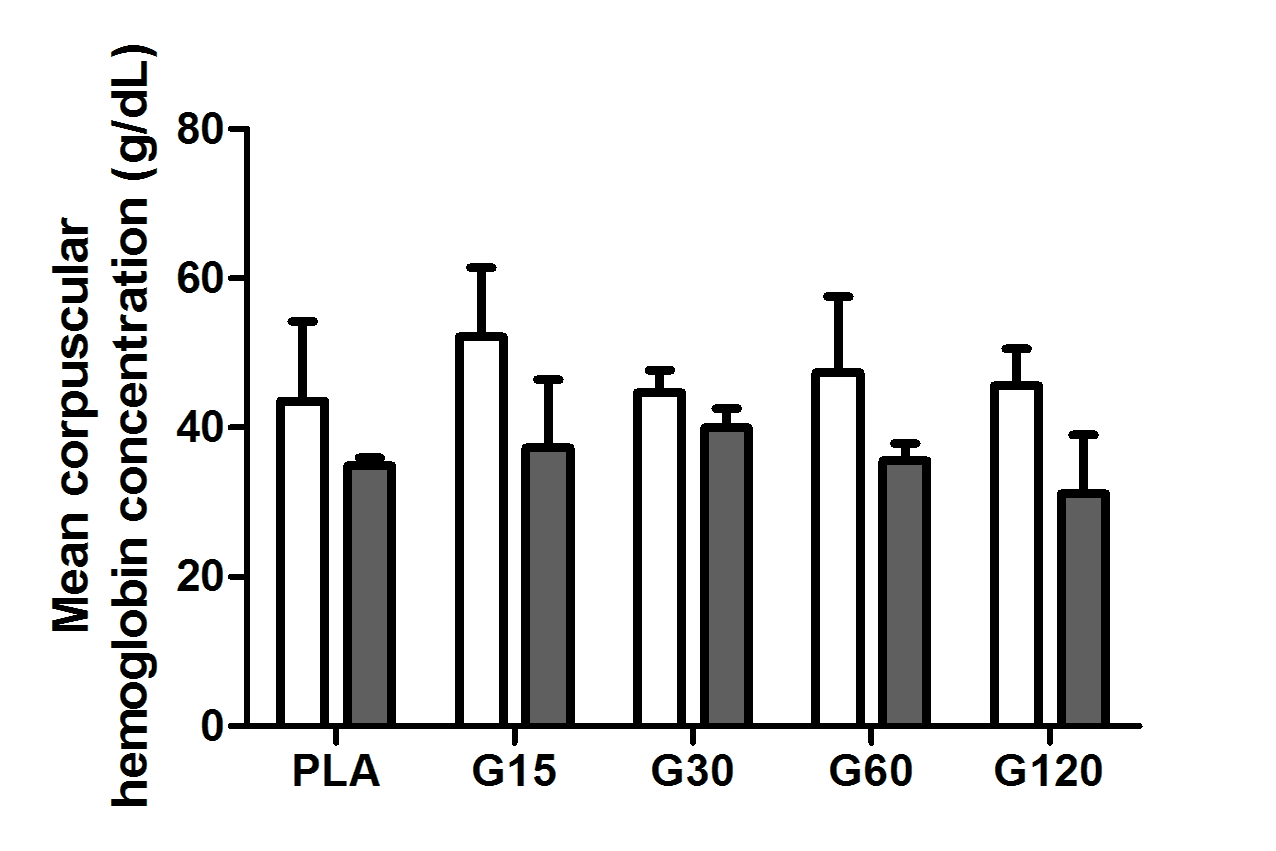

Supplement: Additional file 4 — Effects of different doses of MVIIC on mean corpuscular hemoglobin concentration. After spinal cord injury, controls were injected with sterile water (PLA) and other groups received different doses of MVIIC (15, 30, 60 and 120 pmol). Values represent the means ± SD of six animals at each time. [file 1678-9199-20-15-S4.tiff]

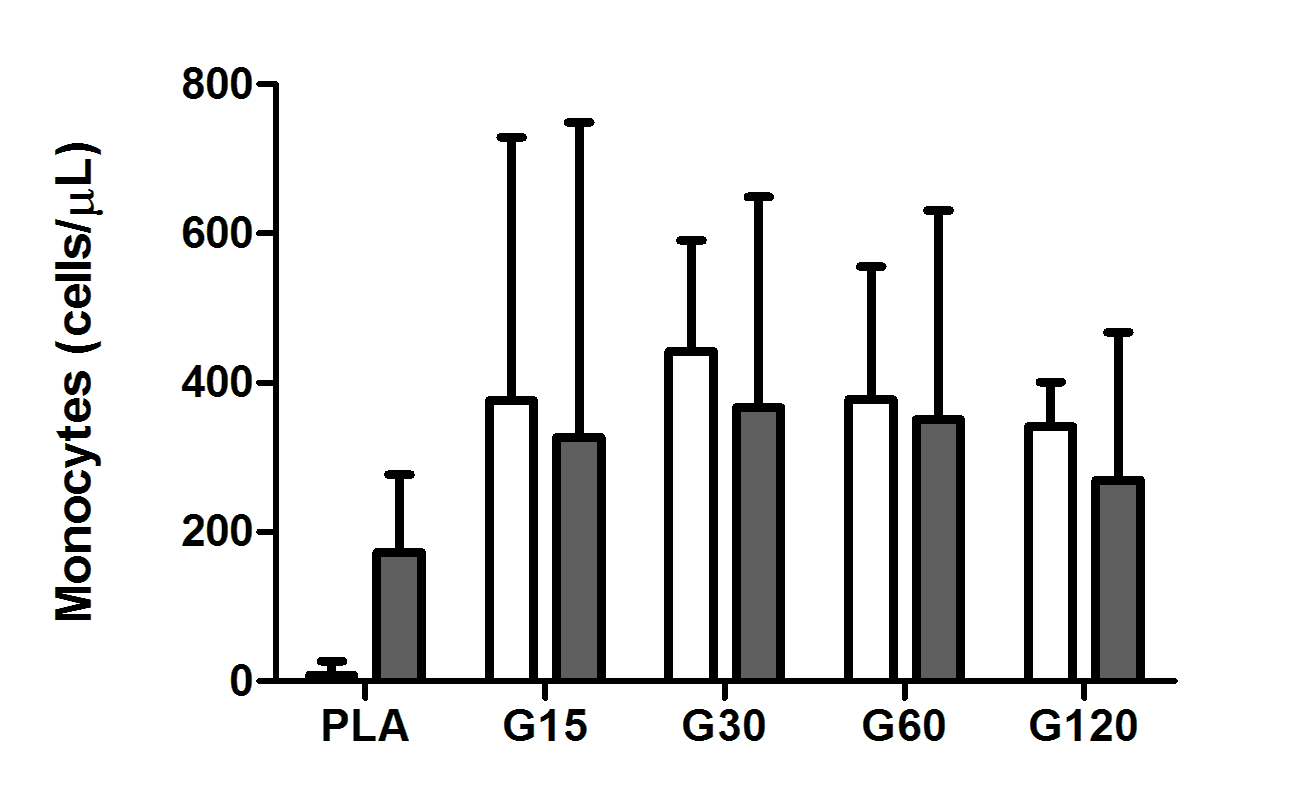

Supplement: Additional file 5 — Effects of different doses of MVIIC on monocyte levels. After spinal cord injury, controls were injected with sterile water (PLA) and other groups received different doses of MVIIC (15, 30, 60 and 120 pmol). Values represent the means ± SD of six animals at each time. [file 1678-9199-20-15-S5.tiff]

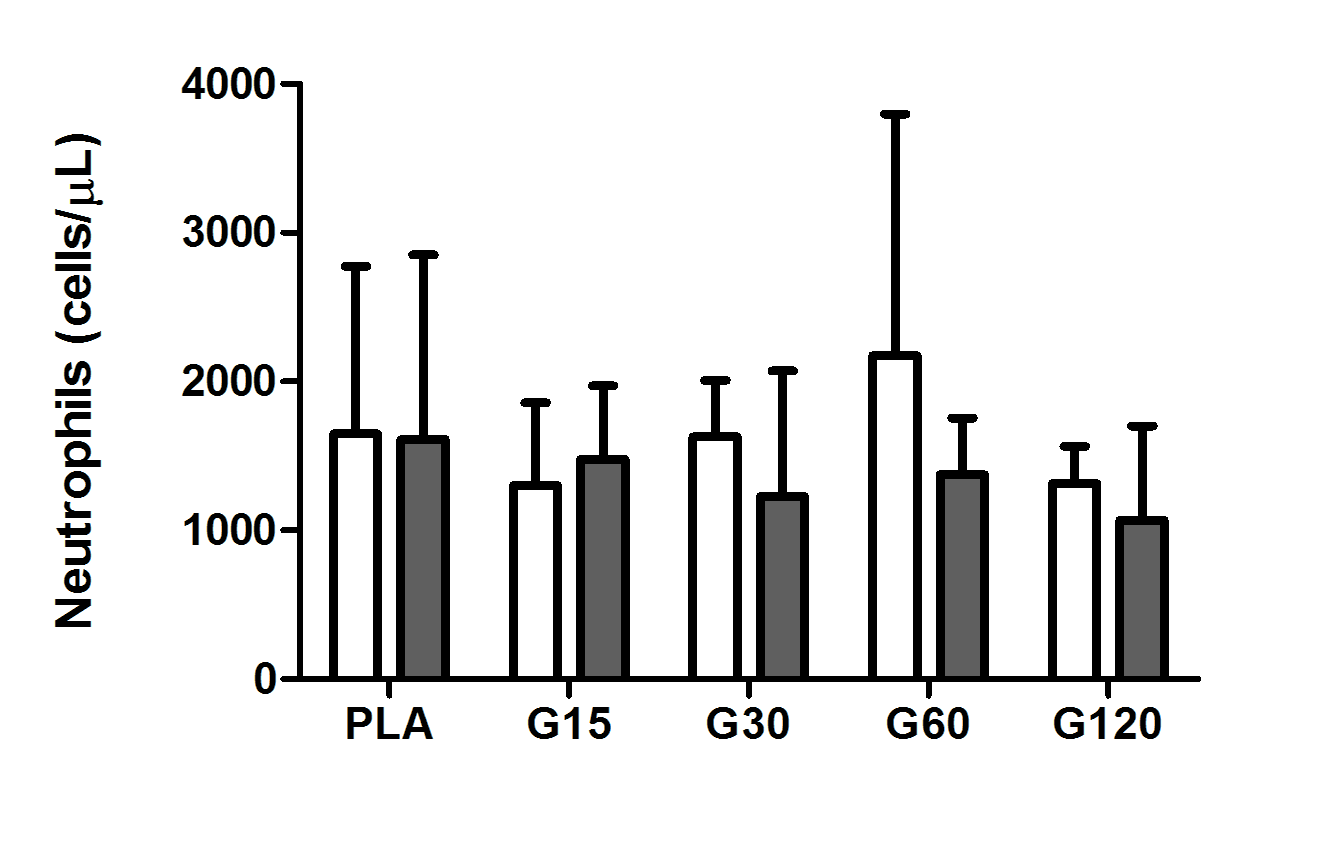

Supplement: Additional file 6 — Effects of different doses of MVIIC on neutrophil levels. After spinal cord injury, controls were injected with sterile water (PLA) and other groups received different doses of MVIIC (15, 30, 60 and 120 pmol). Values represent the means ± SD of six animals at each time. [file 1678-9199-20-15-S6.tiff]

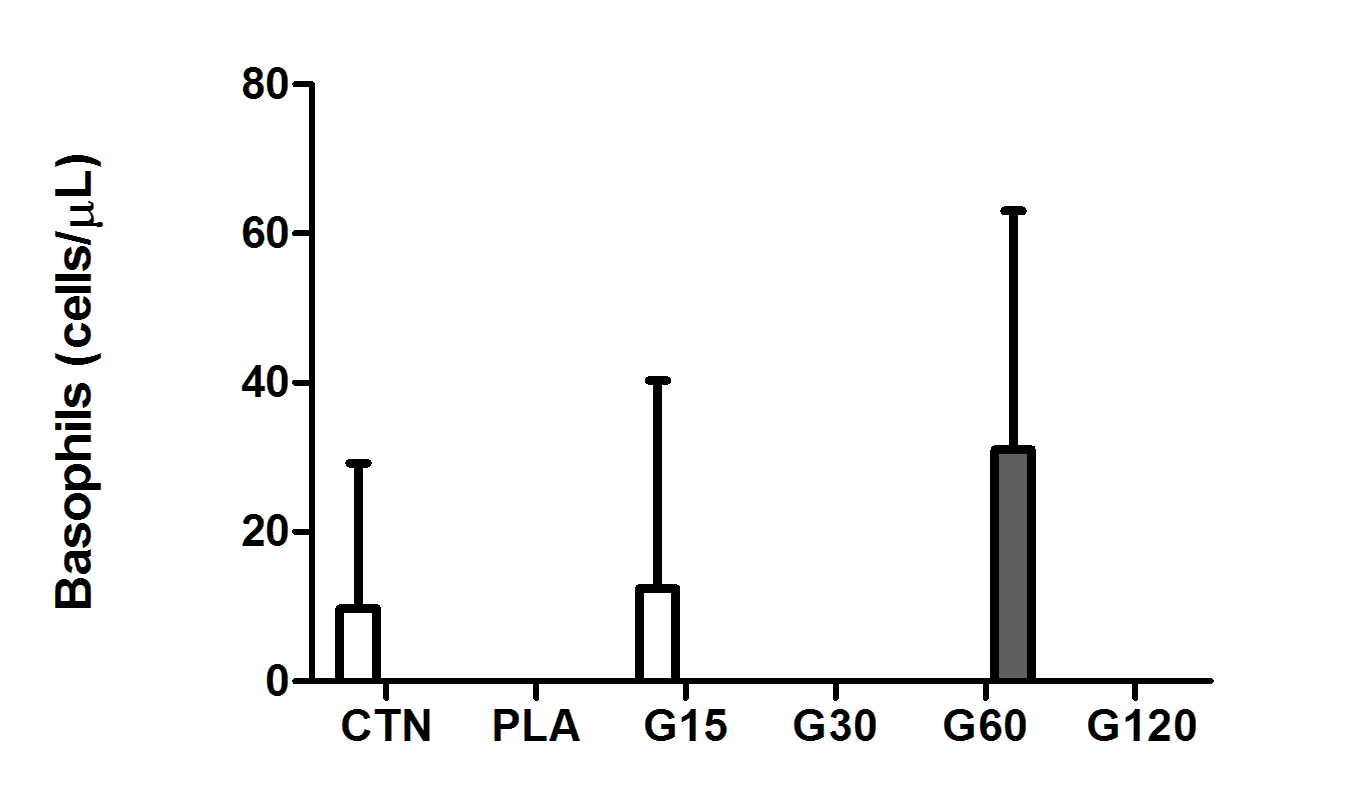

Supplement: Additional file 7 — Effects of different doses of MVIIC on basophil levels. After spinal cord injury, controls were injected with sterile water (PLA) and other groups received different doses of MVIIC (15, 30, 60 and 120 pmol). Values represent the means ± SD of six animals at each time. [file 1678-9199-20-15-S7.tiff]
